# Supplementary material for: Selection for insecticide resistance can promote Plasmodium falciparum infection in Anopheles
Source: PLoS Pathog. 2023 Jun 20;19(6):e1011448. doi: 10.1371/journal.ppat.1011448 (PMC10313043; doi:10.1371/journal.ppat.1011448)
Supplement: S1 Table — (DOCX) [file ppat.1011448.s008.docx]

| **S1 Table: Primer sequences for qRT-PCR.** | | |
| --- | --- | --- |
| *CYP4G17* | F | 5' TGACGGTGGACATTCTGCTC |
|  | R | 5' GTCACACATTTTCATGACAGCCA |
| *CYP4G16* | F | 5' GAAGTTGCGTCGGACGTAAATCTA |
|  | R | 5' GTCTTCGATTTGCGTTGACGTGGTTC |
| *CYP6M2* | F | 5' TACGATGACAACAAGGGCAAG |
|  | R | 5' GCGATCGTGGAAGTACTGG |
| *CYP6P3* | F | 5' TGTGATTGACGAAACCCTTCGGAAG |
|  | R | 5' ATAGTCCACAGACGGTACGCGGG |
| *CYP6Z2* | F | 5' CCACGCAATTGCATTGGTCT |
|  | R | 5' TTCTACGCGCATGGGGAAAC |
